# Impact of Female Obesity on Cumulative Live Birth Rates in the First Complete Ovarian Stimulation Cycle

**DOI:** 10.3389/fendo.2019.00516

**Published:** 2019-08-02

**Authors:** Wen Ding, Fu-li Zhang, Xiao-cong Liu, Lin-li Hu, Shan-jun Dai, Gang Li, Hui-juan Kong, Yi-hong Guo

**Affiliations:** ^1^Reproductive Medicine Center, First Affiliated Hospital of Zhengzhou University, Zhengzhou, China; ^2^Henan Key Laboratory of Reproduction and Genetics, First Affiliated Hospital of Zhengzhou University, Zhengzhou, China

**Keywords:** BMI, polycystic ovary syndrome, ovarian stimulation, cumulative live birth rates, IVF/ICSI outcome

## Abstract

**Background:** Female overweight/obesity has been reported to be associated with compromised pregnancy outcomes in fresh embryo transfer cycles. It is unclear whether the cumulative live birth rate (CLBR) is adversely affected after all viable embryos are transferred from the first ovarian stimulation cycle.

**Objectives:** To investigate whether the CLBR was compromised in obese women.

**Method:** A total of 9,772 young women underwent their first IVF/ICSI cycles from January 2012 to October 2017. Pregnancy outcomes were compared according to female BMI.

**Results:** Among 1,671 women with polycystic ovary syndrome (PCOS), those with a BMI ≥ 28 kg/m^2^ had a lower cumulative clinical pregnancy rate (CCPR) and CLBR during the first complete ovarian stimulation cycle. Additionally, the pregnancy loss rate was increased in this group, although the difference was not significant. Among the 8,101 women without PCOS, the CCPR and CLBR of obese patients was also significantly decreased, and this group also showed increased pregnancy loss rates. Moreover, overweight women also had a decreased CLBR.

**Conclusions:** Female obesity adversely affected the CLBR after utilizing the viable embryos from first oocytes retrieval.

## Introduction

Polycystic ovary syndrome (PCOS), the most common endocrine disorder in women, is the main cause of ovulatory infertility. The underlying mechanisms include hyperandrogenism, hypersecretion of luteinizing hormone (LH), hyperinsulinemia, and altered secretion of adipokines (1). As reported previously, the proportion of obese women with PCOS could be as high as 50% (2). Obesity affects reproductive function mainly by disturbing neuroendocrine and ovarian function, leading to anovulation (1). Moreover, female obesity is related to the development of PCOS (3). A retrospective study by Provost MP et al. with a total of 22,317 donor oocyte IVF cycles showed that pregnancy outcomes were worse in women with a high BMI (4). Rittenberg et al. found that overweight/obese women had significantly increased miscarriage rates (RR = 1.31, *P* < 0.0001), along with significantly decreased clinical pregnancy rates (RR = 0.9, *P* < 0.0001) and live birth rates (RR = 0.84, *P* < 0.0002) (5). Similar results were found for women with PCOS in China (6). However, a few reports showed the opposite results. For example, a prospective study from nine hospitals in China reported that, among women with PCOS, the ongoing pregnancy rate was similar for women with a high BMI (7). Female overweight/obesity has been associated with compromised pregnancy outcomes in fresh embryo transfer cycles, while the effect of female BMI on the cumulative live birth rate (CLBR) is unclear. Thus, the present study was performed to determine whether female obesity has an adverse effect on the CLBR.

## Materials and Methods

### Patients

We retrospectively enrolled 1,671 Chinese patients diagnosed with PCOS by the Rotterdam criteria and 8,101 non-PCOS patients who received infertility treatment at the Reproductive Medicine Center of the First Affiliated Hospital of Zhengzhou University and Henan Key Laboratory of Reproduction and Genetics of the First Affiliated Hospital of Zhengzhou University from January 2012 to October 2017. Patients were included if they had received their first fresh IVF/ICSI cycles with autologous oocytes. All included female subjects were under the age of 35. The treatment cycles were excluded if donor oocytes or sperm were utilized. Female subjects with a medical history of endometriosis, reproductive malformation, or uterine fibroids were excluded. Couples who received a preimplantation genetic diagnosis or underwent preimplantation genetic screening were excluded. Patients without written informed consent were excluded.

### Dataset

A large cohort study was performed based on electronic medical records. A total of 1,671 Chinese women diagnosed with PCOS by the Rotterdam criteria and 8,101 women without PCOS in our study were stratified into three groups based on the BMI guidelines for the Chinese population (8): normal-weight group (18.5–23.9 kg/m^2^), overweight group (23.9–27.9 kg/m^2^), and obese group (BMI ≥ 28 kg/m^2^).

### IVF Protocol

The patients included in our study were treated with gonadotropin releasing hormone (GnRH) agonists to prevent premature LH surge, and injectable gonadotropins were administered to stimulate follicle growth. We regularly monitored follicle growth by transvaginal ultrasound and the serum estradiol, progesterone and LH levels during the cycle. When at least one follicle had a mean diameter of more than 18 mm, human chorionic gonadotropin (hCG) was given, and oocyte retrieval was performed 36 h later. Fresh embryo transfers were performed on Day 3 or Day 5, and subsequent frozen-thawed transfer was performed through a natural cycle with or without hCG or through an artificial cycle using estradiol. The number of embryos transferred varied from one to three based on the recommendation of the Health Ministry of China and the requests of patients.

Indications for the freeze-all policy included moderate-severe ovarian hyperstimulation syndrome (OHSS), a high risk of developing moderate-severe OHSS, inadequate endometrial thickness and individual preference. Risk factors of developing moderate-severe OHSS included diagnosis of mild OHSS, low body mass, young women with good ovarian reserve, an estradiol level >3,500 pg/ml, and more than 15 oocytes retrieved. The decision regarding the freeze-all policy was made by experienced clinicians depending on clinical symptoms, laboratory parameters and the risk factors listed above. The classification of OHSS was based on the criteria described by Golan and Weissman (9).

### Outcomes

The primary outcome was the CLBR, while secondary outcome was the cumulative clinical pregnancy rate (CCPR). Clinical pregnancy was achieved when the intrauterine gestational sac was recognized by ultrasonography after embryo transfer and a positive serum β-hCG concentration was found. Live birth was defined as at least one live infant born after 24 weeks of gestation, and the CLBR was defined as the percentage of live births per woman during the first complete stimulation cycle, including fresh transfer or subsequent frozen-thawed embryo transfer after the first oocyte retrieval. Miscarriage was defined as intrauterine pregnancy loss before 24 weeks of gestation. The dropout rates were the proportions of patients who failed to achieve a live birth and discontinued the treatment with remaining frozen embryos.

According to the findings from published reports (10, 11), underweight women have a higher risk of miscarriage and a decreased LBR in fresh embryo transfer cycles, and female overweight/obesity is also correlated to compromised pregnancy outcomes. Therefore, normal-weight patients were used as the reference group for comparison.

### Statistical Analysis

Statistical analyses were performed using SPSS 17.0 software (IBM corp., Armonk, NY). Continuous variables, such as female age, are presented as the median (IQR) and compared via the Kruskal–Wallis test, while categorical variables, such as the CCPR and CLBR, are presented as frequencies (percentages) and were compared via the Chi-square test. The difference of proportions between groups was compared using Bonferroni correction.

For the first complete ovarian stimulation cycle, multivariate logistic regression was performed to evaluate the impact of female BMI on pregnancy outcomes for 1,671 PCOS and 8,101 non-PCOS patients. The results are expressed as ORs with 95% confidence intervals. Additionally, adjusted odds ratios (aORs) were obtained by fitting regression models with female age, male age, method of fertilization (IVF or ICSI), total doses of gonadotropins and number of oocytes retrieved. A *P*-value <0.05 was considered significant.

## Results

**Figure 1:**
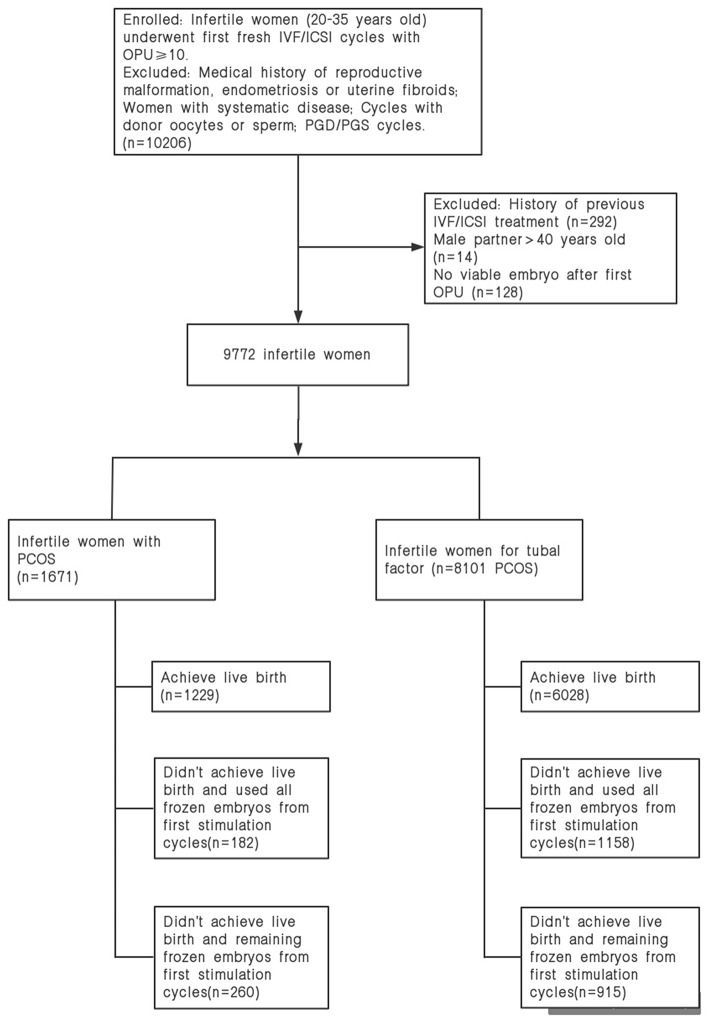
Flow diagram of patient selection.

A total of 1,671 women with PCOS and 8,101 non-PCOS patients were enrolled in our study (Figure 1). All patients were stratified into the normal-weight, overweight or obese group by their BMI. Table 1 presents the basic and clinical characteristics for all included patients. The total doses of gonadotropins increased significantly as female BMI increased (*P* < 0.001). Among patients without PCOS, the number of oocytes yield was statistically different between the normal weight and overweight patients (*P* = 0.013); otherwise, no significant differences were observed for women with PCOS among the three BMI groups. Besides, obese women had fewer available embryos and cryopreserved embryos compared to the normal weight group. No differences were observed in the cacellation rates, moderate-severe OHSS rates, proportion of ICSI, and the sum of transferred embryos among the three BMI groups (Table 1).

**Table 1:** Baseline characteristics and IVF/ICSI outcomes of 9772 infertile women.

	**Normal weight**	**Overweight**	**Obese**	***P***
**Patients with PCOS (*****n*** **= 1,671)**	806	613	252	
Female age (years)^a^	27 (4)	28 (5)	28 (4)	0.022
Male age (years)^a^	28 (5)	29 (6)	29 (6)	0.010
ICSI (%)	152 (18.8%)	100 (16.3%)	39 (15.5%)	NS
Total Gn dose (IU)^a^^,^^b^^,^^c^	1500 (787.5)	1925 (1125)	2356.2 (1378.1)	<0.001
No. of retrieved oocytes	18 (9)	19 (9)	18 (10)	NS
No. of available embryos^c^	7 (5)	7 (5)	6 (4)	0.022
No. of cryopreserved embryos^c^	6 (5)	6 (5)	6 (5)	0.016
No. of transferred embryos	2 (2)	2 (2)	3 (2)	NS
Cancellation rate (%)	424 (52.6%)	318 (51.8%)	114 (45.2%)	NS
Moderate-severe OHSS (%)	90 (11.2%)	61 (9.9%)	21 (8.3%)	NS
**Patients for tubal factor (*****n*** **= 8,101)**	5812	1802	487	
Female age (years)^a^^,^^c^	28 (5)	29 (6)	29 (6)	<0.001
Male age (years)^a^^,^^c^	29 (6)	30 (6)	30 (6)	<0.001
ICSI (%)	1634 (28.1%)	466 (25.8%)	113 (23.2%)	0.019
Total Gn dose (IU)^a^^,^^b^^,^^c^	1650 (837.5)	2050 (1037.5)	2362.5 (1237.5)	<0.001
No. of retrieved oocytes^a^	15 (7)	16 (7)	16 (7)	0.013
No. of available embryos^c^	6 (4)	6 (4)	6 (4)	0.041
No. of cryopreserved embryos^c^	5 (4)	5 (4)	5 (4)	0.024
No. of transferred embryos	2 (2)	2 (2)	2 (2)	NS
Cancellation rate (%)	1742 (29.9%)	491 (27.2%)	131 (26.9%)	0.044
Moderate-severe OHSS (%)	476 (8.2%)	128 (7.1%)	28 (5.7%)	NS

*Data are presented as the median (IQR) or frequency (percentage). P-values are calculated using the Chi-square test for proportions and the Kruskal–Wallis test for medians among the three BMI groups. Letter a,b,c indicated significant difference between groups*.

a *P: Comparison between Normal weight and Overweight patients*.

b *P: Comparison between Overweight and Obese patients*.

c *P: Comparison between Normal weight and Obese patients*.

Table 2 shows the pregnancy outcomes of women with PCOS during the first complete ovarian stimulation cycle. Compared to the normal-weight group, obese women with PCOS (BMI ≥28 kg/m^2^) had 52% lower odds of cumulative clinical pregnancy (aOR: 0.48, 95%CI 0.34–0.68) and 50% lower odds of cumulative live birth (aOR: 0.50, 95%CI 0.37–0.68) after adjusting for confounding factors. Moreover, the odds of miscarriage were increased in this group (aOR: 1.26, 95%CI 0.80–1.97), although no significant difference was found. However, overweight women with PCOS (BMI 23.9–27.9 kg/m^2^) had 28% lower odds of cumulative clinical pregnancy (aOR: 0.72, 95%CI 0.54–0.97) and 32% lower odds of cumulative live birth (aOR: 0.68, 95%CI 0.53–0.86) compared to the normal-weight group. Additionally, the odds of miscarriage were increased (aOR: 1.25, 95%CI 0.91–1.73), although the difference was not significant.

**Table 2:** Multivariate logistic regression for pregnancy outcomes in the first complete ovarian stimulation cycles.

**Patients with PCOS (*n* = 1,671)**	**Normal weight**	**Overweight**	**Obese**	***P***
Cumulative CPR^c^	696 (86.3%)	504 (82.2%)	190 (75.4%)	<0.001
OR (95%CI)	REF	**0.73 (0.55–0.98)**	**0.48 (0.34–0.69)**	
aOR (95%CI)	REF	**0.72 (0.54–0.97)**	**0.48 (0.34–0.68)**	
Cumulative LBR^a^^,^^c^	632 (78.4%)	435 (70.9%)	162 (64.3%)	<0.001
OR (95%CI)	REF	**0.67 (0.53–0.85)**	**0.49 (0.36–0.67)**	
aOR (95%CI)	REF	**0.68 (0.53–0.86)**	**0.50 (0.37–0.68)**	
Miscarriage (<24weeks)	91 (13.1%)	82 (16.3%)	30 (15.8%)	NS
OR (95%CI)	REF	1.29 (0.93–1.78)	1.25 (0.79–1.95)	
aOR (95%CI)	REF	1.26 (0.91–1.75)	1.26 (0.80–1.97)	

*Data are presented as frequencies (percentages), crude ORs (95%CIs), or adjusted ORs (95%CIs). Adjusted confounding factors including female age, male age, total Gn dose, method of insemination, and oocytes yield*.

a *P: Chi-square test for proportions between Normal weight and Overweight patients*.

c *P: Chi-square test for proportions between Normal weight and Obese patients*.

*The bold values indicated lower odds of cumulative clinical pregnancy and live birth, and the difference was significant*.

Table 3 indicates that obese women without PCOS (BMI ≥28 kg/m^2^) had significantly decreased odds of cumulative clinical pregnancy (aOR: 0.67, 95%CI 0.54–0.84) and cumulative live birth (aOR: 0.60, 95%CI 0.49–0.73) after utilizing viable embryos from the first oocyte retrieval cycle, when compared to normal weight women. Meanwhile, obese women had higher odds of miscarriage (aOR: 1.53, 95%CI 1.14–2.06). Similarly, overweight (BMI 23.9–27.9 kg/m^2^) women had lower odds of cumulative live birth (aOR: 0.85, 95%CI 0.76–0.96) along with higher odds of miscarriage (aOR: 1.23, 95%CI 1.03–1.46), whereas the cumulative clinical pregnancy rates were slightly lower than those of the reference group, without a significant difference.

**Table 3:** Multivariate logistic regression for pregnancy outcomes in the first complete ovarian stimulation cycles.

**Patients for tubal factor (*n* = 8,101)**	**Normal weight**	**Overweight**	**Obese**	***P***
Cumulative CPR^b^^,^^c^	4794 (82.5%)	1457 (80.8%)	365 (74.9%)	<0.001
OR (95%CI)	REF	0.89 (0.78–1.03)	**0.63 (0.51–0.78)**	
aOR (95%CI)	REF	0.93 (0.81–1.06)	**0.67 (0.54–0.84)**	
Cumulative LBR^a^^,^^b^^,^^c^	4402 (75.7%)	1309 (72.6%)	317 (65.1%)	<0.001
OR (95%CI)	REF	**0.85 (0.75–0.96)**	**0.59 (0.49–0.72)**	
aOR (95%CI)	REF	**0.85 (0.76–0.96)**	**0.60 (0.49–0.73)**	
Miscarriage (<24weeks)^a^^,^^c^	518 (10.8%)	191 (13.1%)	58 (15.9%)	0.002
OR (95%CI)	REF	**1.24 (1.04–1.49)**	**1.56 (1.16–2.10)**	
aOR (95%CI)	REF	**1.23 (1.03–1.46)**	**1.53 (1.14–2.06)**	

*Data are presented as frequencies (percentages), crude ORs (95%CIs), or adjusted ORs (95%CIs). Adjusted confounding factors including female age, male age, total Gn dose, method of insemination, and oocytes yield*.

a *P: Chi-square test for proportions between Normal weight and Overweight patients*.

b *P: Chi-square test for proportions between Overweight and Obese patients*.

c *P: Chi-square test for proportions between Normal weight and Obese patients*.

*The bold values indicated lower odds of cumulative live birth and higher odds of miscarriage, and the difference was significant*.

Among the 9,772 infertile patients who underwent at least one cycle of embryo transfer, 1,175 failed to achieve a live birth and discontinued treatment with the remaining frozen embryos. Results indicated that obese women had higher dropout rates from treatment than the normal weight group. The dropout rates of overweight/obese patients were higher (9.5 vs. 9.4%) in the second embryo transfer cycles, which continuously increased in the subsequent embryo transfer cycles, especially for obese women (Table 4).

**Table 4:** Patients lost to follow-up with remaining frozen embryo(s) ≥ 1 in the first complete ovarian stimulation cycles.

**Embryo transfer cycles after first OPU**	**Normal weight**	**Overweight**	**Obese**	***P***
Patients underwent 2nd ET cycles)	2,879	1061	365	
Discontinued the 2nd ET cycles	238 (7.6%)	112 (9.5%)	38 (9.4%)	NS
Patients underwent 3rd ET cycles)	1250	511	145	
Discontinued the 3rd ET cycles^b^^,^^c^	244 (16.3%)	101 (16.5%)	56 (27.8%)	<0.001
Patients underwent 4th ET cycles)	492	212	56	
Discontinued the 4th ET cycles	224 (31.3%)	123 (36.7%)	39 (41.1%)	NS

*All enrolled patients underwent at least one cycle of embryo transfer. Patients lost to follow-up were those who discontinued infertility treatment without achieving live birth after first OPU, while there were remaining frozen embryos*.

*Patients discontinued the 4th ET cycles included those who underwent at least three repeated embryo transfer cycles*.

b *P: Comparison between Overweight and Obese patients*.

c *P: Comparison between Normal weight and Obese patients*.

## Discussion

Our study retrospectively included 9,772 infertile women who underwent their first complete ovarian stimulation cycles. Obese women with PCOS had a decreased CCPR and CLBR and an increased miscarriage rate compared to normal-weight women. Moreover, obese women without PCOS also had a decreased CCPR and CLBR and a higher risk of pregnancy loss than normal weight patients. These results suggested that female obesity was an independent risk factor for the CLBR. Overweight patients had a decreased CLBR and an increased miscarriage rate as well.

### Female Obesity and Reproductive Outcomes

Female overweight/obesity has been associated with morphological changes in oocytes and phenotypic changes and metabolic abnormalities in embryos (12). A previous review by Talmor A also mentioned that the quality of oocytes and embryos could be affected due to the alterations in the endocrine environment related to body weight (3). Moreover, the endometrium is vital for successful implantation, and Bellver et al. revealed differences in endometrial gene expression during the window of implantation among obese patients, especially for those with PCOS (13). Moreover, obesity was thought to be an independent risk factor for endometrial polyps (14). All of these changes are underlying mechanisms for the decreased assisted reproductive technology (ART) success rate in overweight/obese women.

A large retrospective cohort study of 239,127 fresh autologous IVF cycles from the United States demonstrated that obesity adversely influenced IVF outcomes, causing decreased odds of implantation (OR: 0.96, 95%CI 0.93–0.98), clinical pregnancy (OR: 0.83, 95%CI 0.75–0.92), and live birth (OR: 0.75, 95%CI 0.67–0.83) and higher odds of pregnancy loss (OR: 1.67, 95%CI 1.33–2.09) among patients with ovulation disorders (15). Another review also showed that increased female BMI was associated with a decreased ART success rate (5). Obese patients had a decreased LBR in fresh embryo transfer cycles, which was probably due to increased gonadotropin utilization during ovarian hyperstimulation as reported (16). The total doses of gonadotropins utilized were significantly increased for both overweight and obese women (Table 1). Considering the subsequent frozen-thawed embryo transfer cycles from first oocyte retrieval, obese women had a decreased CCPR and CLBR and an increased miscarriage rate; and even though the miscarriage rate increased as female BMI increased among women with PCOS, no significant difference was observed, probably due to the limited sample size of obese patients (Tables 2, 3).

### Cumulative Live Birth Rates and Determining Factors

Several retrospective studies demonstrated that female age and the number of oocytes retrieved were important factors for cumulative live birth. Polyzos et al. performed a multicenter study with 15,000 infertile patients, and the results indicated that the CLBR increased progressively along with the oocyte yield, which could be up to 70% when more than 25 oocytes were retrieved (17). Additionally, similar results were observed by Drakopoulos et al. (18). The number of oocytes retrieved was also beneficial for the LBR of infertile patients with advanced age (≥38 years old), but the effect was decreased for women older than 41 years (19). However, the CLBR in freeze-all cycles was comparable to that of conventional fresh/frozen embryo transfer cycles, whereas the former resulted in a higher CLBR than the latter in blastocyst transfer cycles (20). However, the evidence to demonstrate the impact of female BMI on the CLBR was limited. The present study aimed to explore the effect of female BMI on the CLBR.

Our findings demonstrated that female obesity had a negative impact on the CLBR. Women with PCOS had a significantly decreased CLBR (*P* < 0.001) compared to the normal weight patients, and the included sample size resulted in 97.6% power to detect the difference in the power analysis (21). Accordingly, the CLBR was statistically decreased in obese women compared to the control group (*P* < 0.001), and the power was 99.4% for women without PCOS. Meanwhile, the CLBR was compared between groups using Bonferroni adjustment, and the type I error was adjusted to be 0.0167.

Meanwhile, our study revealed that obese women had increased dropout rates after repeated embryo transfer cycles (Table 4). Koning et al. reported that overweight/obese women had higher costs during their infertility treatment and decreased success rates of achieving a live birth, which were probably correlated with increased dropout rates (22).

### Lifestyle Modifications and Pregnancy Outcomes

Maternal obesity resulted in higher risks of gestational diabetes (GDM), gestational hypertension and pre-eclampsia (23). Additionally, more than half of overweight/obese women gained more weight than recommended during gestation, leading to increased risks of perinatal complications, neonatal adverse outcomes and long-term consequences for maternal and offspring health (24). Regarding the neonatal outcomes, a retrospective study revealed that the normal birth weight (NBW) and fet al macrosomia rates of singletons were increased when the parents had a high BMI (25). Moreover, maternal obesity was also linked to fet al overgrowth, stillbirth, and congenital anomalies (23).

For overweight/obese women who desire to become pregnant, weight loss through lifestyle modification is strongly recommended before infertility treatment. A weight loss of 5–10% from baseline was associated with improved metabolic dysfunction and reproductive function (26, 27), and a previous study conducted by Legro et al. demonstrated that obese women with PCOS who made lifestyle changes before infertility treatment had higher odds of live birth (RR 2.5; 95%CI, 1.3–4.7; *P* = 0.01) than those who sought prompt infertility treatment (28). Nevertheless, the drawback of weight loss through lifestyle changes is weight regain; however, long-term behavioral counseling to provide dietary or activity recommendations is not widely available (27). What's more, the effect of weight management on the assisted reproduction outcomes were still uncertain (29).

The present study supported that female obesity was independent risk factor of the CLBR; meanwhile, obese women had continuously increased dropout rates in subsequent embryo transfer cycles, compared to normal weight women. Considering that a high proportion of women with PCOS were obese, recommendations should be given about pre-pregnancy weight loss through lifestyle modification and gestational weight gain (GWG).

### Strengths and Limitations

Our study revealed that female obesity had a detrimental impact on the CLBR, especially for women with PCOS; Moreover, infertile women with obesity had higher dropout rates during treatment. However, there are several limitations of our study. First, our conclusions were limited due to the retrospective design involving a single medical center. Secondly, the miscarriage rate was calculated only once for each patient, which probably resulted in underestimated pregnancy loss rates. Finally, all enrolled patients underwent ovarian stimulation with a GnRH agonist protocol, which weakens the generalizability of the findings. Therefore, the conclusions need to be interpreted cautiously.

In summary, female obesity was an independent risk factor of the CLBR for the first complete ovarian stimulation cycle. The CLBR of obese patients was significantly decreased in contrast to the normal weight patients after utilizing the viable embryos from first oocytes retrieval. We assumed that this result was likely due to detrimental effect on the quality of oocytes, potential of embryos and endometrial receptivity.

## Ethics Statement

This study was approved by the Institutional Review Board and Ethics Committee of the First Affiliated Hospital of Zhengzhou University (Scientific Research-2018-LW-007), and written informed consent was obtained from all participants.

## Author Contributions

All authors listed have made a substantial, direct and intellectual contribution to the work, and approved it for publication.

### Conflict of Interest Statement

The authors declare that the research was conducted in the absence of any commercial or financial relationships that could be construed as a potential conflict of interest.
